# When to Feed Grain to Horses

**Published:** 1881-09

**Authors:** 


### WHEN TO FEED GRAIN TO HORSES.

Horses are provided with an unusually large development of the salivary glands, and an enormous quantity of saliva is secreted during the eating of a feed of grain or hay. This copious supply of saliva is amply sufficient to moisten and dilute the food, so that it can be digested perfectly without the help of water. Water is absorbed by the coats of the stomach and enters the blood with such rapidity that a thirsty horse will drink in two minutes considerably more water than the stomach will contain at one time, and the water begins to pass off through the kidneys in such a case after the lapse of a very few minutes. So that, knowing these facts, one may reasonably infer that a horse may be watered a few minutes before feeding with more advantage than soon afterward, because in the former case the water has been absorbed before the food is swallowed, and digestion cannot be interfered with by the presence of too much water in the stomach, as might happen in the latter case. The best practice is that usually followed, namely, to give the horse a very little water on starting out to work after feeding in the morning; to water on coming in at noon and in the evening, before unharnessing and feeding. This gives time for the absorption of the water before the food enters the stomach.—*Rural New Yorker.*

---

Cheap Tinware.—Now, a word or two about cheap tinware. You needn't expect to get a dish-pan for 20 cents that's worth 60. They don't grow, and if they ever did the tree is dead and the seed lost. When you buy a pan for 20 cents, depend upon it, it's only worth 20 cents. The iron of which it is made is of an inferior quality, brittle and rotten. It won't stand bending. The tin with which it is covered is nearly all lead, and there's very little of it, anyway; and it will wear off and the iron rust through in a few weeks, while a 60-cent pan, made of good charcoal iron and covered with pure tin and plenty of it, will last you for years.—*The Enterprise.*

---

Barley Water.—A doctor says: I have found this useful in private practice to keep up the strength of a patient. A cupful of barley in two quarts of water, allowed to boil two hours. About an hour after it is on the fire add a dozen stoned raisins; strain before serving. It ought to be cooked in a porcelain-lined vessel, or it is apt to discolor.

